# Functional validation of a novel *STAT3* ‘variant of unknown significance’ identifies a new case of STAT3 GOF syndrome and reveals broad immune cell defects.

**DOI:** 10.1093/cei/uxaf005

**Published:** 2025-01-21

**Authors:** Joseph Mackie, Daniel Suan, Peter McNaughton, Filomeen Haerynck, Michael O’Sullivan, Antoine Guerin, Cindy S Ma, Stuart G Tangye

**Affiliations:** Garvan Institute of Medical Research, Darlinghurst, NSW, Australia; School of Clinical Medicine, Faculty of Medicine and Health, UNSW Sydney, Kensington, NSW, Australia; Garvan Institute of Medical Research, Darlinghurst, NSW, Australia; Clinical Immunogenomics Research Consortium of Australasia (CIRCA), Darlinghurst, NSW, Australia; Clinical Immunogenomics Research Consortium of Australasia (CIRCA), Darlinghurst, NSW, Australia; Queensland Paediatric Immunology and Allergy Service, Queensland Children’s Hospital, South Brisbane, Australia; Department of Pediatric Pulmonology, Infectious Diseases and Immunology, Ghent University Hospital, Ghent, Belgium; Primary Immunodeficiency Research Lab, Centre for Primary Immunodeficiency Ghent, Ghent University Hospital, Ghent, Belgium; Clinical Immunogenomics Research Consortium of Australasia (CIRCA), Darlinghurst, NSW, Australia; Department of Clinical Immunology and PathWest, Fiona Stanley Hospital, Murdoch, WA, Australia; Garvan Institute of Medical Research, Darlinghurst, NSW, Australia; School of Clinical Medicine, Faculty of Medicine and Health, UNSW Sydney, Kensington, NSW, Australia; Garvan Institute of Medical Research, Darlinghurst, NSW, Australia; School of Clinical Medicine, Faculty of Medicine and Health, UNSW Sydney, Kensington, NSW, Australia; Clinical Immunogenomics Research Consortium of Australasia (CIRCA), Darlinghurst, NSW, Australia; Garvan Institute of Medical Research, Darlinghurst, NSW, Australia; School of Clinical Medicine, Faculty of Medicine and Health, UNSW Sydney, Kensington, NSW, Australia; Clinical Immunogenomics Research Consortium of Australasia (CIRCA), Darlinghurst, NSW, Australia

**Keywords:** immunogenetics, immunodeficiency, autoimmunity, immuno-dysregulation, hypogammaglobulinemia

## Abstract

**Introduction:**

Signal transducer and activator of transcription 3 (STAT3) orchestrates crucial immune responses through its pleiotropic functions as a transcription factor. Patients with germline monoallelic dominant negative or hypermorphic *STAT3* variants, who present with immunodeficiency and/or immune dysregulation, have revealed the importance of balanced STAT3 signaling in lymphocyte differentiation and function, and immune homeostasis. Here, we report a novel missense variant of unknown significance in the DNA-binding domain of STAT3 in a patient who experienced hypogammaglobulinemia, lymphadenopathy, hepatosplenomegaly, immune thrombocytopenia, eczema, and enteropathy over a 35-year period.

**Methods:**

*In vitro* demonstration of prolonged STAT3 activation due to delayed dephosphorylation, and enhanced transcriptional activity, confirmed this to be a novel pathogenic *STAT3* gain-of-function variant. Peripheral blood lymphocytes from this patient, and patients with confirmed STAT3 Gain-of-function Syndrome, were collected to investigate mechanisms of disease pathogenesis.

**Results:**

B cell dysregulation was evidenced by a loss of class-switched memory B cells and a significantly expanded CD19^hi^CD21^lo^ B cell population, likely influenced by a skewed CXCR3^+^ T_FH_ population. Interestingly, unlike STAT3 dominant negative variants, cytokine secretion by activated peripheral blood STAT3 GOF CD4^+^ T cells and frequencies of Treg cells were intact, suggesting CD4^+^ T cell dysregulation likely occurs at sites of disease rather than the periphery

**Conclusion:**

This study provides an in-depth case study in confirming a *STAT3* gain-of-function variant and identifies lymphocyte dysregulation in the peripheral blood of patients with STAT3 gain-of-function syndrome. Identifying cellular biomarkers of disease provides a flow cytometric-based screen to guide validation of additional novel *STAT3* gain-of-function variants as well as provide insights into putative mechanisms of disease pathogenesis.

## Graphical Abstract

**Graphical Abstract:**
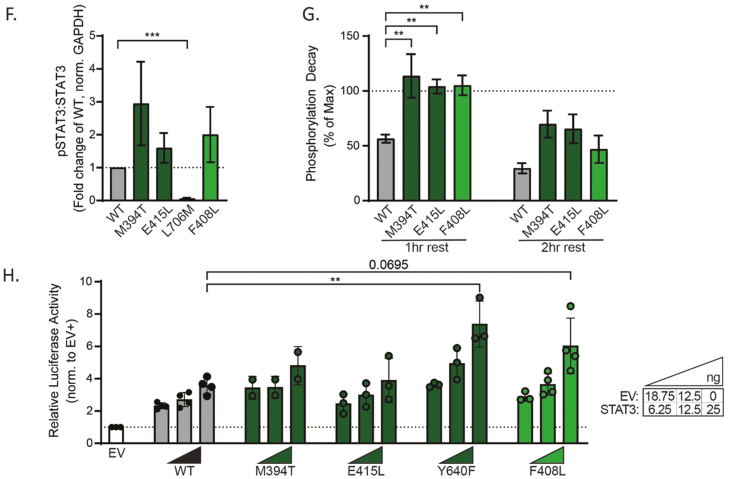

## Introduction

Signal transducer and activator of transcription 3 (STAT3) is a highly pleiotropic transcription factor expressed across all human tissues and implicated in a range of biological processes that underpin health and disease, including response to infectious pathogens, autoimmunity, and malignancy [1, 2]. This pleiotropy arises due to the wide array of ligand/receptor pairs that activate STAT3, including cytokines belonging to the interleukin (IL)-6/gp130 (IL-6, IL-11, IL-27, IL-35, IL-39, cardiotrophin-like cytokine factor 1, oncostatin M, leukemia inhibitory factor, ciliary neurotophic factor), IL-10 (IL-10, IL-19, IL-20, IL-22, IL-24, IL-26, IL-28, IL-29), and common gamma chain (specifically IL-21) families, as well as IL-12, IL-23, and some colony-stimulating factors [1, 3]. STAT3 is also activated by hormones (epidermal growth factor, fibroblast growth factor, growth hormone, insulin-like growth factor), underscoring this transcription factor as a key mediator of cell-to-cell signaling in immune and non-immune pathways [1, 3].

Following the binding of cytokine ligands to cognate receptors, receptor-bound Janus kinases (JAK1/2, TYK2) phosphorylate a canonical tyrosine residue in STAT3 (Tyr705), leading to dimerization and nuclear translocation. Here, activated STAT3 dimers bind a vast array of cis-regulatory motifs across the genome to regulate the transcription of target genes that contribute to cell development, differentiation, and survival [4, 5]. Previous studies have shown that STAT3 directly controls the transcription of crucial immune genes in B cells (*BCL6*, *PRDM1*, *XBP1*) and T_FH_ cells (*BCL6*) in response to IL-21 to establish robust T cell-dependent, B cell-mediated antibody (Ab) responses [6–8]. STAT3 is also critical for inducing *RORC*, a transcription factor necessary for inducing naïve CD4^+^ T cell differentiation into IL-17A-, IL-17F-, IL-22-, and IL-26-secreting T_H_17 cells, which are critical for host defense against fungal infection [9–11], but can also be pathogenic in the setting of autoimmune and inflammatory conditions.

The non-redundant role of STAT3 in cytokine-mediated development, differentiation, and function of human lymphocytes has been unequivocally demonstrated through the identification of rare patients carrying *STAT3* germline heterozygous loss-of-function/dominant negative (LOF/DN) or gain-of-function (GOF) variants, which cause multisystem, life-threatening diseases [12–15]*. STAT3* LOF/DN variants are the most common genetic etiology of hyper-IgE syndrome, presenting early in life with recurrent sinopulmonary infections (*Staph*, *Strep* spp.) resulting in pneumatoceles and/or bronchiectasis, chronic mucocutaneous candidiasis (*Candida albicans*), extremely high levels of serum IgE, atopic dermatitis, and eosinophilia. Affected individuals can also develop a spectrum of non-immunological manifestations such as recurrent bone fractures, craniosynostosis, hyper-extensibility, facial asymmetry, and retention of primary teeth [16]. Conversely, variants encoding a *STAT3* GOF allele cause an early-onset and heterogeneous multisystemic disease—STAT3 GOF Syndrome—most commonly characterized by polyclonal lymphoproliferation, autoimmunity (cytopenias, enteropathy, endocrinopathies e.g. type 1 diabetes mellitus), infectious disease susceptibility (bacterial, viral, and fungal), skin disease (atopic dermatitis), interstitial lung disease, and growth failure [17]. Thus, these rare inborn errors of immunity, affecting ~1 in 100 000 live births, highlight the critical importance of balanced STAT3 signaling in human immunity to maintain homeostasis and prevent infectious disease, autoimmunity, and exaggerated inflammation [18, 19].

Recent advances in next-generation sequencing and improved healthcare access to genetic testing have revolutionized the diagnostic landscape of severe, often early-onset and life-threatening immunological diseases [20, 21]. Expedient genetic diagnoses alleviate unnecessary diagnostic odysseys to curtail considerable patient and economic burden, inform appropriate gene-directed therapies for improved outcomes, and establish molecular mechanisms underlying complex immunological diseases [21]. However, the genetic approach to diagnosing idiopathic diseases requires robust functional validation of candidate variants to confirm causality. This often cannot be immediately ascertained by the combination of clinical presentation, family segregation, allele frequency in the general population, and *in silico* predictive modeling tools [22–25]. Therefore, variants identified in patients without definitive causality, referred to as variants of unknown significance (VUS), have emerged as a considerable challenge to achieving definitive genetic diagnoses for individuals with inborn errors of immunity [26].

Here, we describe a case of a male patient who experienced a considerable 35-year diagnostic odyssey, initially diagnosed with idiopathic common variable immunodeficiency (CVID). Gene sequencing at age 35 years revealed an unreported VUS affecting the DNA-binding domain (DNABD) of *STAT3*. While this patient was included in a large study of the natural history of STAT3 GOF syndrome [17], limited information was provided regarding his extensive clinical history. Furthermore, data establishing the identified *STAT3* VUS was pathogenic or describing cellular defects have not been presented. For these reasons, we now describe the extensive *in vitro* functional testing necessary to validate this variant as GOF, define a common biochemical mechanism of hypermorphic STAT3 transcriptional activity, and identify peripheral biomarkers of disease in STAT3 GOF Syndrome.

## Materials and methods

### Ethics statement

Peripheral blood was collected from STAT3 GOF Syndrome patients; healthy donor (HD) buffy coats were purchased from the Australian Red Cross Blood Service. This study was approved by the Sydney Local Health District RPAH Zone Human Research Ethics Committee and Research Governance Office, Royal Prince Alfred Hospital, Camperdown, NSW, Australia (Protocols X16-0210/LNR/16/RPAH/257 and X16-0210 & 2019/ETH06359; and Protocol X20-0177 & 2020/ETH00998). Written informed consent was obtained from participants, and experiments using human samples were conducted in accordance with local regulations and with the approval of the IRBs of corresponding institutions.

### Primary cells

Peripheral blood mononuclear cells (PBMCs) were collected from lithium-heparinized HD or patient blood samples by Ficoll-Paque (Merck) density gradient fractionation. Cells were cryopreserved in vapor-phase liquid nitrogen until used. T-blasts were produced by culturing 10^4^ PBMCs in ImmunoCult-XF T cell Expansion Media with ImmunoCult Human CD3/CD28/CD2 T cell Activator (Stemcell) and IL-2 (10 ng/ml) for 2 weeks.

### Transient overexpression of STAT3 variants

Site-directed mutagenesis was performed on pCMV6 plasmids containing C-terminal Flag-tagged *STAT3* cDNA (NM_139276, OriGene) using the Q5 Site-Directed Mutagenesis Kit (New England Biolabs) to incorporate variants of interest and transformed into competent *E. coli* (New England Biolabs) for amplification. Plasmids were transiently transfected into A4 *STAT3*^−/−^ (provided by Jean-Laurent Casanova, Institute Imagine, Paris) or HEK293T (ATCC #CRL3216) cell lines using the Lipofectamine 30 000 transfection reagent (Thermo Fisher) supplemented with Opti-MEM (Thermo Fisher) for 24 h before further treatment or lysis was performed.

### Cell lysis and immunoblotting

Transiently transfected A4 *STAT3*^−/−^ cells (24 h) or patient-derived T-blasts (2 weeks) were washed in phosphate buffered saline (PBS) before being lysed in 50mM pH 7.4 Tris-HCl, 150 mM NaCl, 0.5% Triton X-100, 2 mM ethylenediaminetetraacetic acid (EDTA), 0.1 mM dithiothreitol (DTT), and protease inhibitor cocktail (Roche). A Bradford assay (Bio-Rad) was used to load an equal protein quantity from lysate into 10% polyacrylamide gel before wet transfer to a polyvinylidene difluoride membrane (Bio-Rad). Membranes were blocked in Intercept Protein-Free Blocking Buffer (LI-COR) for 1 h at room temperature and probed with unconjugated IgG monoclonal antibodies (mAbs) specific for Flag epitope tag (Cell Signaling Technologies, M2), STAT3 (Cell Signaling Technologies, D3Z2G), pY705 STAT3 (Cell Signaling Technologies, 3E2), GAPDH (Santa Cruz, 6C5) overnight at 4°C. Anti-mouse-IgG (IRDye680RD) and anti-rabbit-IgG (IRDye800CW) secondary antibodies (LI-COR) were incubated on membranes for an hour to visualize protein expression using the Odyssey CLx Imager (LI-COR). Images were analyzed with ImageStudio software (LI-COR).

### Assessment of kinetics of STAT3 phosphorylation

Following 24 h of STAT3 plasmid transfection, A4 *STAT3*^−/−^ cells were stimulated with (100 ng/ml) or without (serum-free Roswell Park Memorial Institute media [RPMI]) IL-6 (PeproTech) for 15 min. Dephosphorylation was assessed by washing cells with serum-free RPMI and incubating the cells at 37°C until 1 h and 2 h post initial stimulation. Cell activation was quenched with ice-cold PBS and lysed with whole cell lysis media.

### Luciferase assay

Transcriptional activity of variants was assessed using the Dual-Luciferase Reporter Assay System (Promega, E1980) following the manufacturer’s instructions. A4 STAT3^−/−^ cells were co-transfected with a reporter vector (pGL 4.47) containing five copies of the luciferase reporter gene, *Luc2P*, downstream of the sis-inducible element, and a constitutively expressed vector containing *Renilla* luciferase (pGL 4.74). Expression vectors containing wildtype (WT) STAT3, variant STAT3, or empty expression vector were also co-transfected and incubated for 24 h before being mock stimulated or stimulated with 50 ng/ml IL-6/IL-6Rα for a further 24 h. Cells were then lysed before firefly, and Renilla luciferase substrate was sequentially introduced and luminescence read on a CLARIOstar plate reader. The ratio of firefly to Renilla luminescence was used to calculate relative luciferase activity due to STAT3.

### Deep immunophenotyping

Cryopreserved PBMCs were analyzed with a high dimensional flow cytometric panel as previously described [27]. Lymphocyte subpopulation frequencies and mean fluorescent index (MFI) were determined using the following conjugated mAbs: anti-CD45RA BUV395, anti-CD8 BUV496, anti-CD21 BUV563, anti-CXCR5 BUV615, anti-PD1 BUV661, anti-CD20 BUV805, anti-CXCR3 BV421, anti-IgD BV480, anti-CD3 BV570, anti-VαQ TCR BV605, anti-CD10 BV650, anti-Vγδ TCR BV711, anti-CD19 BV750, anti-CD161 BV786, anti-CD27 BB515, anti-CD57 BB630, anti-IgG BB660, anti-CD127 BB700, anti-CD56 BB790, anti-CD25 BYG586, anti-CCR6 PE-CF594, anti-IgA1/IgA2 PE-Cy5, anti-CCR7 PE-Cy7, anti-CD4 APC, anti-IgM APC-R700, and anti-Vα7.2 TCR APC-Cy7. Stained cells were run on the BDFACS Symphony A5 flow cytometer.

### Isolation and functional characterization of patient T cells

Naïve (CD45RA^+^CCR7^+^) and memory (CD45RA^-^CCR7^+/−^) CD4^+^ T cells were isolated by florescence-activated cell sorting following the exclusion of Tregs (CD4^+^CD25^hi^ CD127^−^). Sorted cells (3 × 10^4^) were cultured in 96-well plates with TAE (T cell activation and expansion) beads (mAb against CD2, CD3, CD28), with or without (T_H_0) T_H_-polarizing cytokine conditions (T_H_1: 50 ng/ml IL-12; T_H_9: 100 U/ml IL-4, 2.5 ng/ml TGFβ; T_H_17: 50 ng/ml IL-1β, 50 ng/ml IL-6, 50 ng/ml IL-21, 50 ng/ml IL-23, 2.5 ng/ml TGFβ) for 5 days, as previously described [10]. Culture supernatants were collected for cytokine production with a Cytometric Bead Array (BD Biosciences), and cells were restimulated (100 ng/ml PMA, 750 ng/ml ionomycin) for 4 h, treated with Brefeldin A (10 μg/ml) for 2 h, before intracellular cytokine staining was performed. Saponin permeabilized cells were stained with mAbs including anti-IL-4 PE-Cy7, anti-IL-9 PerCPCy5.5, anti-IL-13 BV421, anti-IL17A APC-Cy7, and anti-IL-17F BV786 and run on a BDFortessa flow cytometer.

## Results

### Case presentation

**Figure 1:**
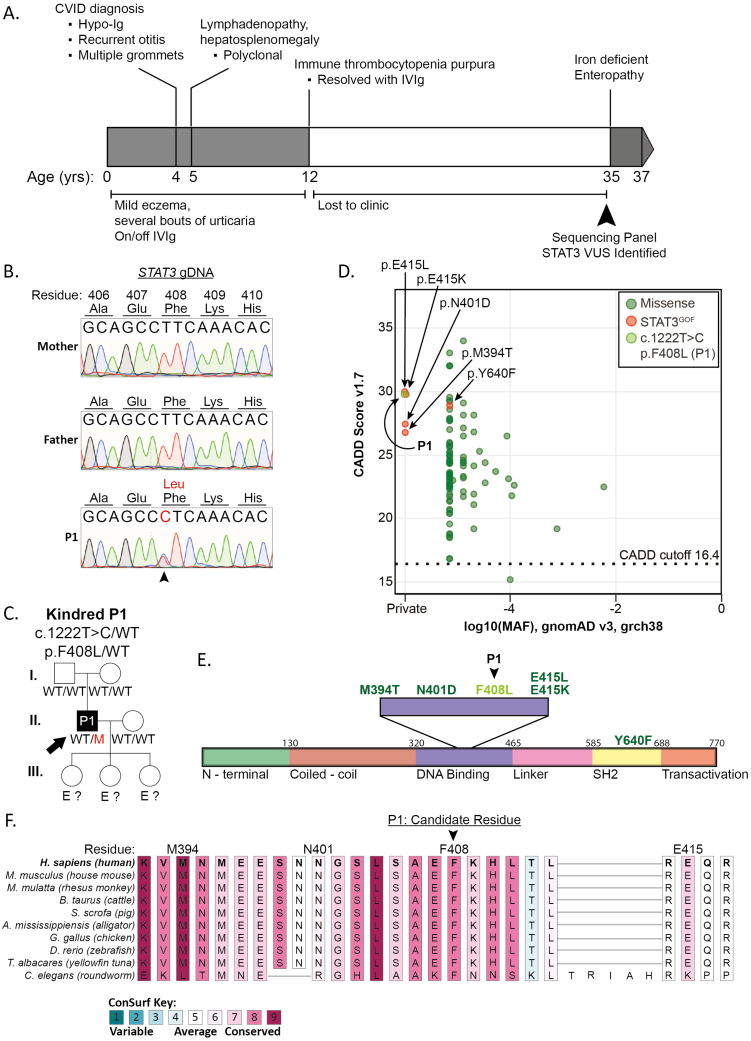
disease course in a patient with idiopathic CVID over a 35-year period with a novel *STAT3* variant. (**A**) Timeline of clinical presentations observed in a patient with CVID. (**B**) Sanger sequencing of gDNA from P1 and their mother and father confirming a novel *de novo* variant in STAT3 (c. 1222T > C, p. F408L). (**C**) Pedigree of the family carrying the novel *STAT3* VUS. (**D**) gnomAD minor allele frequency versus CADD score for the novel variant (P1), the adjacent DNABD GOF variants, and the SH2 GOF variant, p.Y640F. The mutation significance cutoff (99% confidence interval) is represented by the dotted line. (**E**) Functional domain schematic of STAT3 protein highlighting the DNABD region in which novel P1 VUS (F408L) is located. Previously validated STAT3 GOF variants (M394T, N401D, E415K/L, Y640F) are depitcted as dark green. (**F**) Conservation alignment and ConSurf score of DNABD amino acids from 19 representative species through evolution.

The index case (P1) was born to healthy, non-consanguineous, Caucasian parents (parenthood not confirmed genetically). He presented with mild eczematic skin disease, urticaria, and recurrent otitis media requiring multiple grommets during his first year of life (Fig. 1A). Testing at age 4 years revealed very low serum Ig leading to a diagnosis of CVID and commencement of IVIg replacement. Lymphadenopathy and hepatosplenomegaly developed at 5 years of age, with a lymph node biopsy demonstrating polyclonal lymphoproliferation. Immune thrombocytopenia developed in adolescence during a period of IVIg but responded following the re-commencement of IVIg. Over the next two decades, the patient was lost to follow-up but reported minimal complications until representing at age 35 years with enteropathy and iron deficiency (Fig. 1A).

A targeted inborn error of immunity (IEI) gene sequencing panel identified a novel variant in *STAT3* (c. 1222T > C), resulting in a missense phenylalanine to leucine substitution at amino acid 408 (p. F408L; Fig. 1B). The variant was absent from the proband’s parents and was thus *de novo* (Fig. 1B and C). The variant affected the DNABD of STAT3 and was located near four previously reported missense variants known to be causal for STAT3 GOF Syndrome (M394T, N401D, E415L, E415K; Fig. 1E). Despite comparable biochemical properties of phenylalanine and leucine as amino acids (non-polar, identical molecular weight), the variant was private to the family, being absent from the gnomAD population database (Fig. 1D). The variant had a combined annotation-dependent depletion (CADD) score of 29.7, exceeding the gene-specific mutation significance cutoff (16.4, 99% confidence interval), and being comparable to pathogenic DNABD variants M394T, N401D, E415L, and E415K, as well as a pathogenic Src homology 2 domain variant previously reported in STAT3 GOF patients and various neoplasms (Y640F) [17, 28, 29] (Fig. 1D and E). Alignment of amino acid sequences containing this novel F408L variant and known variants from representative species indicate high evolutionary conservation of the STAT3 DNABD region in vertebrates, which is supported by high ConSurf scores at these affected residues, suggesting evolutionary constraint (Fig. 1F). Taken together, *in silico* assessment of this novel VUS and patient disease presentation indicated this variant is likely pathogenic {PM1, PM2, PM6, PP2, and PP3; moderate [American College of Medical Genetics (ACMG) guidelines]} and GOF in nature [17, 23].

#### Functional validation of STAT3 F408L

**Figure 2:**
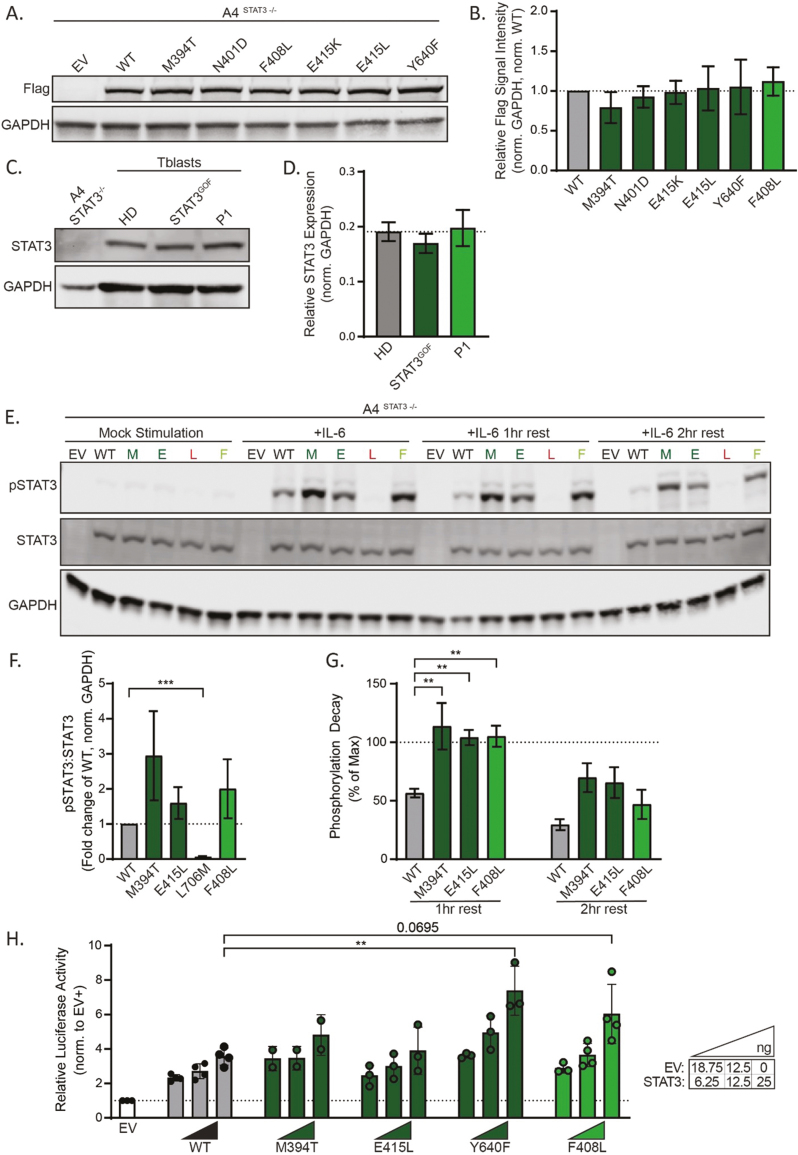
functional validation of a novel *STAT3* missense variant. (**A**, **B**, **E–G**, **H**) A4^*STAT3*−/−^ cells were transfected with pCMV6 EV or containing flag epitope-tagged WT or variant *STAT3* for 24 h. (A) Lysed cells were analyzed for STAT3 expression in a representative western blot, quantified in (B) with each bar representing the intensity of flag signal (normalized to GAPDH), and normalized to WT for three independent experiments. Statistical significance was determined by a one-way ANOVA with Dunnett’s post-test. (**C**, **D**) Lysates of patient-derived blasting T cells from HDs, a STAT3 GOF patient (p.T716M), and P1 were analyzed in a representative western blot (C) to assess endogenous STAT3 protein quantity. (D) represents STAT3 signal intensity normalized to GAPDH in two independent experiments (*n* of HD = 7). Statistical significance was determined by a one-way ANOVA with Dunnett’s post-test. (E) Representative western blot to assess the kinetics of STAT3 activation (pSTAT3) and dephosphorylation. Twenty-four hours following transfection with EV or vector encoding WT, p.M394T (M), p.E415L (E), p.L706M (L), or p.F408L (F) STAT3, A4^*STAT3*−/−*-*^ cells were cultured in SFM or 100 ng/ml IL-6 for 15 min before being washed in SFM and rested to 1- and 2-h timepoints. Maximum phosphorylation fold change to WT after 15 min is shown in (F) with normalization to total STAT3 and GAPDH from four independent experiments. Percent decay from maximum activation in each WT or variant allele is graphed across four independent experiments in (G) with pSTAT3 normalization to STAT3 and GAPDH. Statistical significance was determined by one-way ANOVA with Dunnett’s post-test (**P < 0.01, ***P < 0.001). (H) A4^*STAT3*−/−^ cells were co-transfected with combinations of EV, WT, and patient variant STAT3 pCMV6 vectors in addition to a firefly luciferase vector containing the M67SIE STAT3-target promoter and a constitutively expressed Renilla luciferase vector for 24 h before being stimulated with 50 ng/ml sIL-6 for 24 h. Lysed cells were assessed for luciferase activity. The graph represents firefly luminescence normalized to Renilla luminescence normalized to the stimulated EV condition. Statistical significance for 25 ng STAT3 dosages was assessed using a one-way ANOVA with Dunnett’s post-test (*P < 0.05, **P < 0.01).

To investigate the biochemical impact of the *STAT3* VUS, the F408L variant was incorporated into a Flag epitope-tagged pCMV6 STAT3 expression plasmid. First, to test the impact the variant has on the expression of the encoded protein, A4 *STAT3*^−/−^ cell lines were transiently transfected with plasmids encoding WT, previously validated STAT3 GOF variants (M394T, N401D, E415K, E415L, Y640F), or the variant identified in P1 (F408L) and assessed for expression by western blot. STAT3 protein expression was unaffected by all validated STAT3 GOF mutations, as well as candidate variant F408L (Fig. 2A and B). This result was confirmed using primary T cell blasts generated from P1 and a patient with the common *STAT3* GOF T716M variant in comparison to HDs, suggesting enhanced protein expression is not a mechanism of GOF at the *STAT3* locus (Fig. 2C and D).

Next, we assessed basal and maximal cytokine-induced STAT3 tyrosine phosphorylation (pTyr) and the kinetics of dephosphorylation due to this VUS in comparison to WT and GOF *STAT3* alleles previously shown to impact pTyr dynamics (M394T, E415L). As a negative control, we also included a previously validated STAT3 LOF/DN (L706M) variant, which severely decreases cytokine-induced STAT3 phosphorylation [4]. In transiently transfected A4 *STAT3*^*−/−*^ cell lines, there was no evidence of constitutive pTyr in the absence of cytokine for any of the tested variants (Fig. 2E). There was a non-significant increase in maximal tyrosine phosphorylation of STAT3 F408L after 15 min of IL-6 treatment compared to WT STAT3 (Fig. 2E–G). Following cessation of IL-6 stimulation by washing cells with serum-free media, the pTyr STAT3 signal of M394T, E415L, and P1 variant F408L remained at 100% of maximum activation (as observed at the 15 min time point, data not shown) 1 h after initial stimulation, whilst phosphorylation of WT STAT3 reduced to 50% of maximal at this timepoint, and it was not until the 2-h timepoint when pTyr status of STAT3 GOF variants normalized towards the level of WT STAT3 (Fig. 2F and G). As expected, STAT3 L706M DN protein failed to undergo significant phosphorylation in response to IL-6 (Fig. 2E and F). A luciferase reporter assay demonstrated that, when transfected into A4 *STAT3*^−/−^ cells, the STAT3 F408L VUS in P1 resulted in significantly increased basal transcriptional activity at the *STAT3* M67SIE promoter in the absence of any exogenous cytokine stimulation (Supplementary Fig. 1A). Increased transcriptional activity of the STAT3^F408L^ variant was also observed following stimulation of transfected A4 *STAT3*^−/−^ cells with sIL-6 (Fig. 2H). To confirm the STAT3^F408L^ variant exhibited increased transcriptional activity under more physiological conditions, we generated T cell blasts from HDs and P1 and assessed the induction of SOCS3 mRNA in response to IL-21. Under these conditions, the kinetics of *SOCS3* upregulation in HD and STAT3^GOF^ T cells was comparable; however, the level of expression was ~2.5–3-fold greater in P1 T cells compared to HD T cells (Supplementary Fig. 1B). Combined, these data strongly indicate that altered dynamics of STAT3 F408L dephosphorylation result in GOF for transcriptional activity and is likely the cause of immune disease in P1.

#### Aberrant B cell immune phenotype in STAT3 GOF

**Figure 3:**
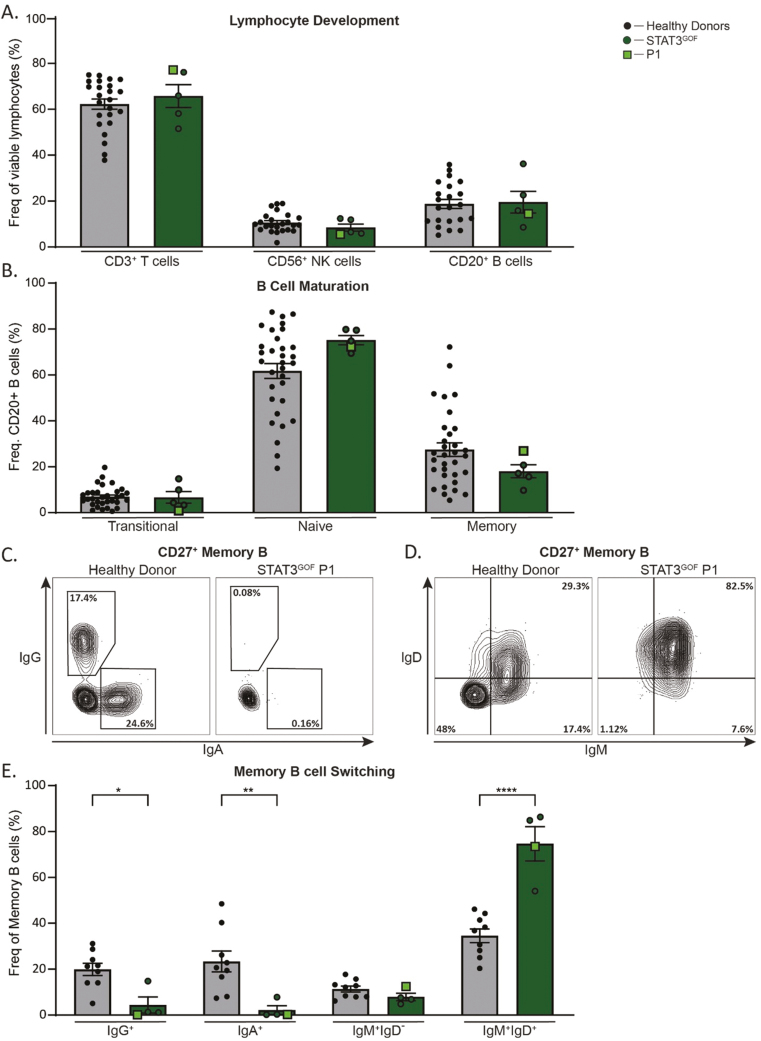
defects in switched memory B cells in STAT3 GOF Syndrome. Flow cytometry of isolated PBMCs from HDs (*n* = 9–26) and STAT3 GOF patients (*n* = 4) was used to determine the frequencies of: (**A**) lymphocytes that were CD3^+^ T cells, CD56^+^ NK cells, and CD20^+^ B cells. (**B**) CD20^+^ B cells that were CD10^+^CD27^-^ transitional, CD10^-^CD27^-^ naïve, or CD10^-^CD27^+^ memory cells. (**C–D**) Representative contour plot of (C) IgG vs IgA or (D) IgD vs IgM gated on CD10^-^ CD27^+^ memory B cells. (**E**) Frequency of CD10^−^CD27^+^ B cells that were switched memory (IgG^+^ or IgA^+^), IgM single positive memory, or unswitched IgM^+^IgD^+^ memory B cells. Statistical significance was calculated using a one-way ANOVA with Dunnett’s post-test, *P < 0.05, **P < 0.01, ***P < 0.0001.

With confirmation of STAT3 GOF Syndrome in P1, we sought to investigate lymphocyte development and function in this patient, and additional patients with confirmed STAT3 GOF variants (*n* = 4; Table 1**).** Despite reports of lymphopenia in STAT3 GOF Syndrome [17], this was not observed in patients included in this study. Indeed, when analyzed by flow cytometry, the frequency of T, NK, and B cells in the patient group was all comparable to the HD cohort (Fig. 3A). B cell maturation was largely intact in the patient group, evidenced by the proportions of transitional, naïve, and memory B cells being similar to HDs (Fig. 3B). However, there was a significant paucity of switched (IgG^+^, IgA^+^), and a concomitant expansion in the frequency of unswitched (IgD^+^ IgM^+^) memory B cells in all STAT3 GOF patients tested (Fig. 3C–E).

**Table 1:** clinical presentation and treatment in five patients with validated STAT3^GOF^ syndrome.

Patient	P1 (novel)	P2	P3	P4	P5
**Age in years** **(gender)**	35 (male)	18 (male)	6 (male)	20 (male)	22 (male)
**STAT3 variant**	p. F408L	p. R152W	p. R246Q	p. T716M	p. T716M
**Clinical features**	Hypo-IgG Recurrent otitis Mild eczema lymphadenopathy and splenomegaly ITP	Bacterial, viral, and fungal infection AIN, AIHA, and ITP Lymphadenopathy and splenomegaly	Evan’s syndrome (neutropenia/ ITP) at age 4 yr	Hypo-IgG	Hypo-IgG Rheumatological manifestations
**Treatment**	No current treatment	Ig replacement, Chronic/pulse steroids, rituximab, rapamycin, antimicrobial prophylaxis	No current treatment	IVIg rapamycin	IVIg Tocilizumab

**Figure 4:**
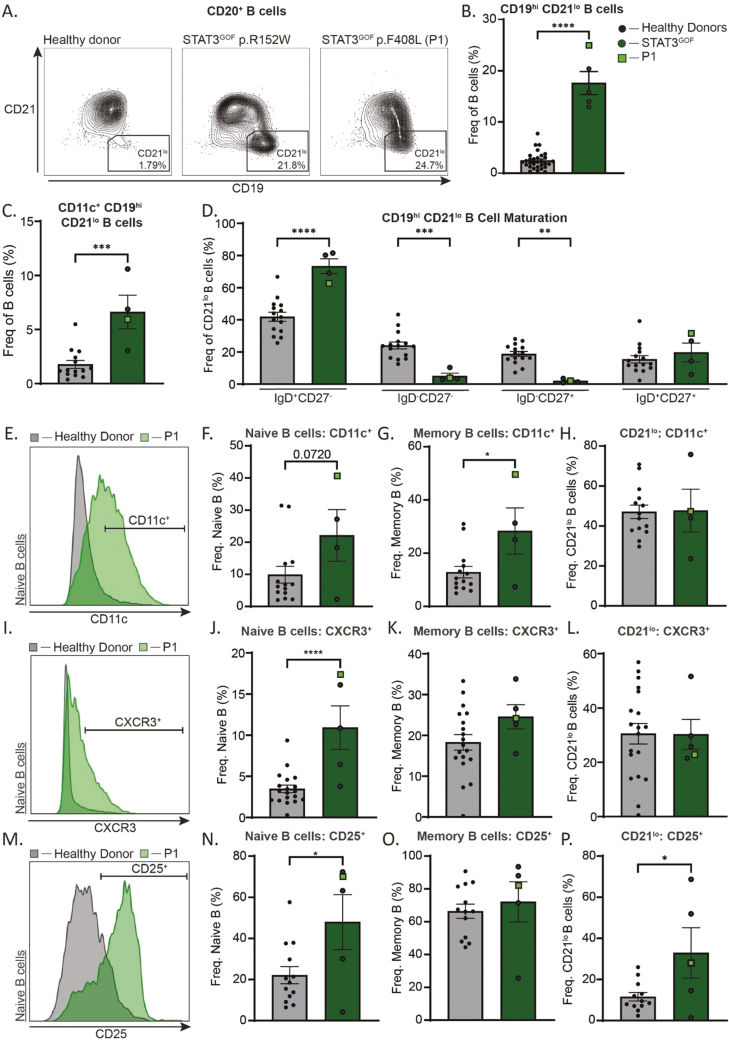
activated naïve atypical B cell phenotype in STAT3 GOF Syndrome. Flow cytometry on isolated PBMCs from HDs (*n* = 12–19) and STAT3 GOF patients (4–5) was used to determine B cell phenotypic features. (**A**) Representative contour plots of CD20^+^ B cells to determine the frequency of CD19^hi^ CD21^lo^ B cells in one HD and two patients with STAT3 GOF Syndrome, graphed in (**B**). (**C**, **D**) Frequencies of CD11c^+^ CD19^hi^ CD21^lo^ cells within the total B cell compartment (C), and of CD19^hi^ CD21^lo^ cells which were IgD^+^ CD27^−^, IgD^−^ CD27^−^, IgD^−^ CD27^+^, and IgD^+^ CD27^+^ (D). (**E**) Representative overlay of CD11c MFI on naïve B cells (CD19^hi^ CD21^lo^ excluded). (**F–H**) Frequencies of naïve (F), memory (G), and CD19^hi^ CD21^lo^ (H) that express CD11c. (**I**) Representative overlay of CXCR3 MFI on naïve B cells (CD19^hi^ CD21^lo^ excluded). (**J–L**) Frequencies of naïve (J), memory (**K**), and CD19^hi^ CD21^lo^ (L) that express CXCR3. (**M**) Representative overlay of CD25 MFI on naïve B cells (CD19^hi^ CD21^lo^ excluded). (**N–P**) Frequencies of naïve (N), memory (O), and CD19^hi^ CD21^lo^ (P) that express CD25. Statistical significance was calculated using a one-way ANOVA with Dunnett’s post-test, *P < 0.05, **P < 0.01, ***P < 0.001, ****P < 0.0001.

CD19^hi^CD21^lo^ B cells, also termed atypical B cells, are increased in patients with autoimmune conditions such as systemic lupus erythematosus, Sjogren’s syndrome, and rheumatoid arthritis, and have been suggested to contribute to disease [30]. A marked enrichment in the frequency of CD19^hi^CD21^lo^ B cells, often comprising up to 20% of the total B cell population, and CD19^hi^CD21^lo^CD11c^+^ activated B cells was also consistently observed for all STAT3 GOF patients (Fig. 4A–C). These atypical B cells were largely unswitched and exhibited a naïve (IgD^+^CD27^-^) phenotype when compared to atypical (CD19^hi^CD21^lo^) B cells from HD (Fig. 4D). This indicates a clear defect in differentiation of this B cell subset, in addition to impaired class switching of the typical (CD21^+^) memory B cell compartment, in STAT3 GOF patients. Although proportions of atypical B cells expressing the integrin CD11c amongst the total B cell population were increased in STAT3 GOF (Fig. 4C), levels of expression of CD11c on CD19^hi^CD21^lo^ B cells were similar for STAT3 GOF patients and HD (Fig. 4H). However, CD11c was expressed on a significantly higher proportion of both naïve and memory B cells (Fig. 4F and G), and at elevated levels on naïve B cells compared to corresponding B cells from HDs (Fig. 4E).

Another feature of CD19^hi^CD21^lo^ B cells is increased expression of the inflammatory chemokine receptor CXCR3 [31]. This was also found to be highly upregulated on naïve B cells from STAT3 GOF patients compared to HDs, while the expression on STAT3 GOF CD19^hi^CD21^lo^ and memory B cells was similar to the B cell subsets from HDs (Fig. 4I–L).

Interestingly, the high-affinity component of the IL-2 receptor, CD25, was expressed on naïve B cells from STAT3 GOF patients, but—consistent with our previous studies [32] —not from HDs. However, CD25 expression on memory B cells from patients with STAT3 GOF Syndrome was comparable to memory B cells from HDs (Fig. 4M–O). CD25 was also disproportionally upregulated on CD19^hi^ CD21^lo^ atypical B cells compared to HDs (Fig. 4P). Taken together, these data establish that peripheral B cells in STAT3 GOF Syndrome patients have an ‘activated naïve’ phenotype, with widespread defective differentiation of both atypical CD21^−^ and mature CD21^+^ B cells likely contributing to defective humoral immunity and potentially supporting the accumulation of autoreactive clones.

#### Phenotype and function of STAT3 GOF CD4^±^ T cells

**Figure 5:**
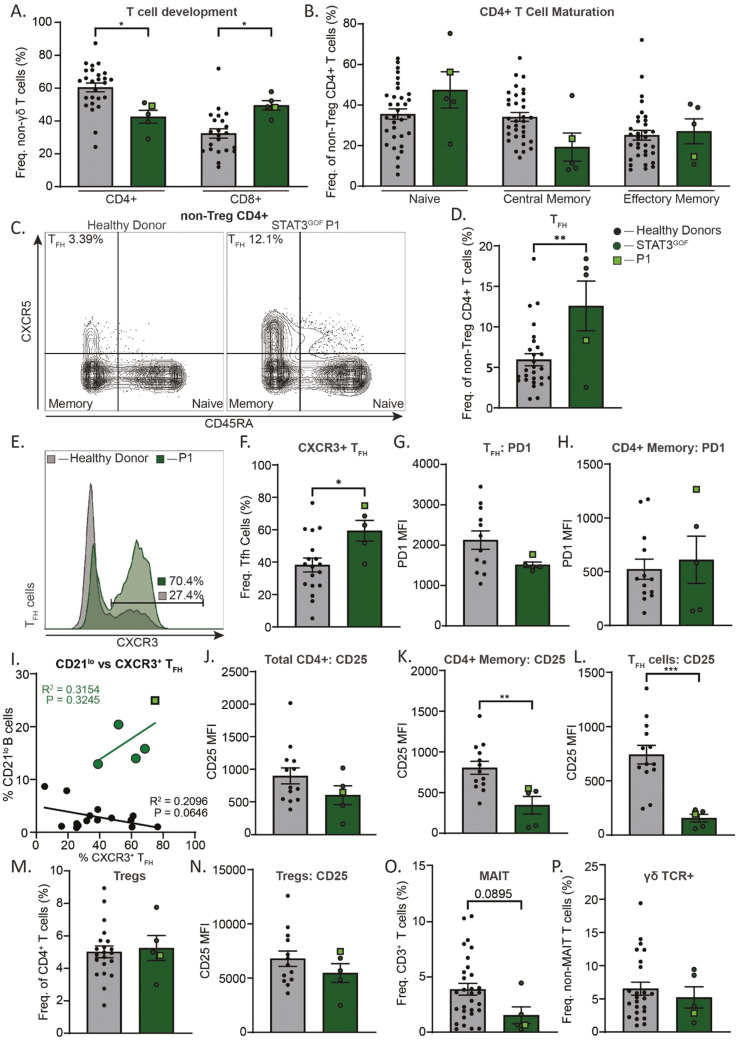
CD4^+^ T cell dysregulation in STAT3 GOF Syndrome. Flow cytometry was used on PBMCs to analyze phenotypic differences between HDs (*n* = 13–37) and STAT3 GOF Syndrome patients (*n* = 5). (**A**) Frequency of CD4^+^ and CD8^+^ cells from non-γδ TCR CD3^+^ T cells. (**B**) Frequency of naïve (CCR7^+^ CD45RA^+^), central memory (CCR7^+^ CD45RA^−^), and effector memory (CCR7^−^ CD45RA^+^) cells from non-Treg CD4^+^ T cells. (**C**) Representative contour plot of non-Treg CD4^+^ T cells to determine naive (CXCR5^-^ CD45RA^+^), memory (CXCR5^−^ CD45RA^−^), and T_FH_ (CXCR5^+^ CD45RA^−^) cell frequencies in a HD and STAT3 GOF Syndrome P1. Graphed in (**D**). (**E**) Representative histogram overlay of CXCR3 expression on T_FH_ cells from a HD and P1. HD and STAT3 GOF cohort graphed in (**F**). (**G** and **H**) MFI of PD1 gated on T_FH_ cells (G) and on CD4^+^ memory cells (H). (**I**) Scatter plot of CD19^hi^ CD21^lo^ frequency of total B cells against frequency of CXCR3^+^ T_FH_ cells. (**J–L**) MFI of CD25 gated on total CD4^+^ T cells (J), CD45RA^-^ CXCR5^+^ memory CD4^+^ T cells (K), and on CD45RA^−^ CXCR5^+^ T_FH_ cell (L). (**M** and **N**) Frequency of Tregs (CD127^−^ CD25^+^) from total CD4^+^ T cells (M) and MFI of CD25 gated on total Tregs (N). (**O** and **P**) Frequency of MAIT cells (CD161^+^ Vα7.2 TCR^+^) amongst total CD3^+^ T cells (O) and frequency of γδ TCR^+^ cells of non-MAIT T cells (P). Statistical significance of column graphs was calculated using a one-way ANOVA with Dunnett’s post-test, *P < 0.05, **P < 0.01, ***P < 0.001. Simple linear regression was used to create lines of best fit in (I). Goodness of fit indicated with R^2^ value and statistical significance of slope deviation from 0 represented by *P*-value.

High parameter immunophenotyping was also used to identify differences in peripheral T cells in patients with STAT3 GOF Syndrome. Compared to HDs, STAT3 GOF patients had higher frequencies of CD8^+^ T cells as a proportion of total T cells (HD: ~60% CD4:40% CD8; STAT3 GOF [~45% CD4:55% CD8], resulting in an inverted CD4:CD8 ratio (Fig. 5A), as described previously [17, 33]. A previous study of three STAT3 GOF patients reported an enhanced frequency of memory CD4^+^ T cells [34]. While we did not confirm these findings (Fig. 5B), we did observe an expansion of total T_FH_ cells and a skewing of T_FH_ cells towards a CXCR3-expressing T_H_1-like phenotype (Fig. 5C–F). Interestingly, there was a positive linear relationship between the frequency of CXCR3^+^ T_FH_ cells and the frequency of CD21^lo^ B cells in STAT3 GOF patients (Fig. 5I). PD1 is expressed on circulating memory and T_FH_ cells in HDs, with levels often being greater on T_FH_ cells (~gMFI = 2000) than memory cells (gMFI = 500, Fig. 5G and H [35]). Interestingly, expression of PD1 on STAT3 GOF T_FH_ cells tended to be lower compared to T_FH_ cells from HDs; however, this difference was not seen for total memory CD4^+^ T cells (Fig. 5G and H).

To extend our findings observed for STAT3 GOF B cells, we also assessed the expression of CD25 on STAT3 GOF CD4 + T cell subsets. In contrast to B cells, we found a stark reduction in CD25 expression on STAT3 GOF T_FH_ cells (7-fold lower that HDs) and total memory CD4^+^ T cells (3-fold) but only marginally reduced on total CD4^+^ T cells (Fig. 5J–L). The role of Tregs in the autoimmune pathogenesis of STAT3 GOF Syndrome has been disputed over the past decade [14, 15, 17, 34, 36, 37]. Our analyses revealed similar proportions of peripheral Tregs (CD4^+^CD127^-^CD25^hi^) in the STAT3 GOF patients and HDs with only a moderate, non-significant reduction in CD25 expression (Fig. 5M and N). In terms of other CD4 + T cell subsets, mucosal-associated invariant T cells were reduced in 4/5 patients investigated, while gd T cells were comparable to HDs (Fig. 5O and P). These data highlight broad phenotypic CD4^+^ T cell dysregulation in patients with STAT3 GOF Syndrome likely influencing immune pathogenesis.

**Figure 6:**
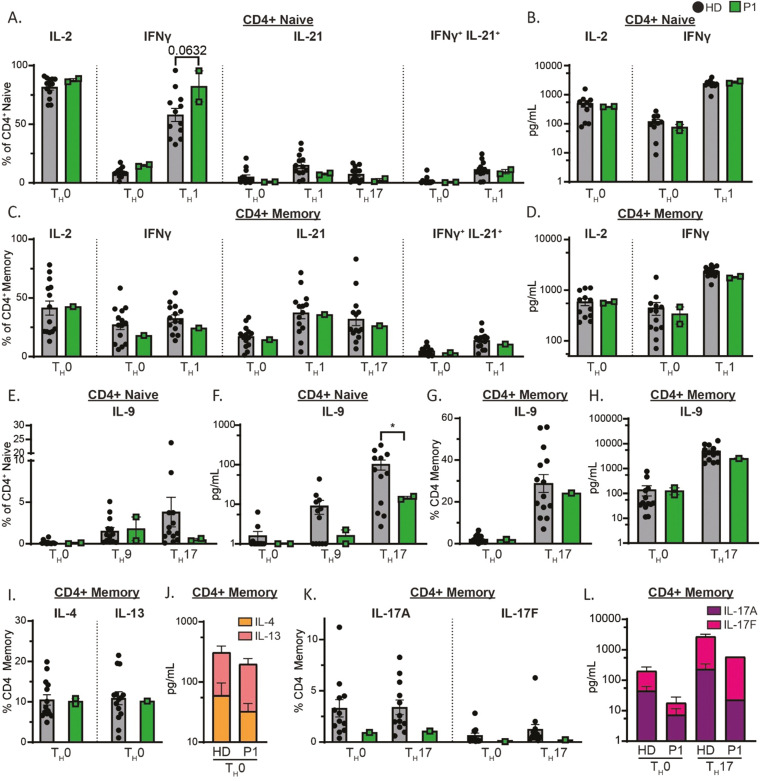
CD4^+^ T cell cytokine expression and secretion in a STAT3 GOF Syndrome patient. Naïve and memory CD4^+^ T cells were sorted by FACS and cultured in T_H_0, T_H_1, T_H_9, or T_H_17 polarizing conditions for 5 days before PMA/Ionomycin/Brefeldin A restimulation and intracellular flow cytometry staining to assess expression of IL-2, IFNγ, IL-21, and IFNγ/IL-21 co-expression (**A**, **C**), IL-9 (**E**, **G**), T_H_2 cytokines (**I**), and T_H_17 cytokines (**K**). Supernatants from cultures were collected and assayed by CBA to detect secretion of IL-2 and IFNγ (**B**, **D**), IL-9 (**F**, **H**), T_H_2 cytokines (**J**), and T_H_17 cytokines (**L**). Statistical significance was calculated using a one-way ANOVA with Dunnett’s post-test, *P < 0.05.

We next sought to evaluate *ex vivo* cytokine secretion as a potential mechanism for autoinflammatory and autoimmune disease presentation in STAT3 GOF patients. Naïve and memory CD4^+^ T cells from HDs and P1 were sorted and cultured with TAE beads with or without T_H_-subset polarizing conditions. IL-2 production and secretion by both naïve and memory CD4^+^ T cells from P1 were comparable to HDs (Fig. 6A–D). There was a trend towards increased IFN-γ production in P1 (*P* = 0.632) following IL-12 (T_H_1) stimulation in naïve CD4^+^ T cells when compared to HD; however, this was normal in secretion in the same culture, as well as in production and secretion in CD4^+^ memory T cells (Fig. 6A–D). IL-21 production was comparable to HDs in all cultures tested (Fig. 6A and C). Further, cells that co-expressed IL-21 and IFN-γ (potent inducers of CD21^lo^ atypical B cells [38, 39]) were both normal in frequency and comparable in sensitivity to induction following T_H_1 polarization, when compared to HDs (Fig. 6A and C).

Production of cytokines that mediate allergic responses (IL-4, IL-9, IL-13), and hence could contribute to atopic skin disease pathogenesis in STAT3 GOF Syndrome, was assessed for CD4^+^ T cells sorted from P1. A trend towards reduced expression and secretion of IL-9 in naïve STAT3 GOF CD4^+^ T cells, particularly in T_H_17 cultures containing STAT3-activating cytokines (IL-6, IL-21, IL-23), but not CD4^+^ memory T cells, was observed (Fig. 6E–H). Production and secretion of T_H_2 cytokines, IL-4 and IL-13, were comparable to HDs in all conditions tested (Fig. 6I and J). A previous report indicated that ~25% of STAT3 GOF patients have an enhanced frequency of IL-17-producing CD4^+^ T cells [17]. However, IL-17A and IL-17F production and secretion by CD4^+^ memory T cells from P1 were found to be non-significantly different from HDs, although there was a consistent trend for reduced production of these cytokines for P1 (Fig. 6I–L). Therefore, across diverse T_H_-stimuli, cytokine production and secretion were largely intact in peripheral naïve and memory CD4^+^ T cells in P1 with the exception of defective IL-9 induction, and possibly reduced Th17 cytokines.

## Discussion

Clinical diagnoses of likely monogenic conditions require thorough validation before putative gene-guided therapies can be considered. Complexity arises when a genetic variant is classified as a VUS, requiring time and resource-intensive functional testing to confirm or exclude pathogenicity. This case study outlines a prolonged 35-year ‘diagnostic odyssey’ from first clinical indication to genetic confirmation of disease, yet a case representative for many patients in the field who often never receive a genetic diagnosis. We outline the requisite steps needed to appropriately classify a candidate VUS in *STAT3* as pathogenic.

*In silico* evaluation of this novel STAT3 F408L variant provided robust evidence, in accordance with gold-standard variant classification ACMG criteria [23], to pursue further functional investigation. In addition to *de novo* inheritance with disease segregation, the absence of P1’s variant from the gnomAD population genetics database and the location of the variant within a region in *STAT3* previously described to harbor GOF variants met three ‘moderate evidence of pathogenicity’ criteria as outlined by ACMG [23]. Further, since *STAT3* has a very low rate of benign missense germline variation with many known missense pathogenic variants, and the high predicted deleteriousness (CADD score) due to high conservation of the affected residue through evolution, this constituted further supporting evidence of pathogenicity and encouraged functional testing.

One mechanism of pathogenicity of germline GOF variants identified in JAK/STAT signaling, specifically STAT1 and STAT6, has been enhanced protein expression, thus facilitating greater signal transduction following cytokine activation [40, 41]. Previous reports in overexpression systems have suggested that this is not the case in *STAT3* GOF alleles [14, 15, 29, 34, 36, 42, 43]. Our study confirmed such observation and extended this to primary T cells from STAT3 GOF patients and HDs, strongly suggesting germline *STAT3* GOF mutations do not induce enhanced auto-induction at its own locus.

STAT3 function is regularly quantified by phosphorylation at a single timepoint. Such an approach fails to detect alterations to the kinetics of activation (min) and regulation (h) of STAT3 signaling and does not assess the function of STAT3 as a transcription factor (i.e. to induce target gene transcription). Thus, we adopted an assay that assessed maximal phosphorylation of STAT3 following cytokine activation, and the kinetics by which STAT3 activation is quenched. This revealed that the STAT3 DNABD F408L variant in P1 did not significantly increase maximal pTyr activation following 15 min of IL-6 stimulation, but instead conferred GOF by delaying dephosphorylation compared to WT STAT3. This prolonged activation resulted in increased transcriptional activity at the M67SIE promoter sequence. Interestingly, neighboring STAT3 GOF mutations M394T and E415L did not exhibit increased transcriptional activity using this luciferase vector, as previously reported [34, 42]. Such *in vitro* experimental nuances should be considered in future validation of novel *STAT3* GOF candidate variants. These functional experiments provided conclusive evidence that the p.F408L STAT3 allele identified in P1 is GOF.

Deep immunophenotyping of patients with complex immunological disease due to monogenic germline variants enables biomarkers to be elucidated and molecular and cellular mechanisms of disease pathogenesis to be unraveled [27, 44, 45]. Leiding *et al*. synthesized clinical and immunological data from 190 patients with STAT3 GOF Syndrome [17], demonstrating significant heterogeneity within common immunophenotypic indications of disease. Therefore, identifying common aspects of immune cell dysregulation could provide unifying mechanisms of disease pathogenesis and support validation of novel VUS.

We identified considerable aberrations to B cell differentiation evidenced by a paucity of Ig class-switched memory B cells and an accumulation of dysregulated CD21^lo^CD19^hi^ B cells and ‘activated naïve’ phenotype B cells in patients with STAT3 GOF Syndrome. The ‘activated naïve’ B cell phenotype with enriched CD21^lo^ B cells has previously been described in the contexts of immunodeficiency, such as CVID [38, 46] and chronic HIV infection [47], autoimmunity such as Hepatitis C virus-associated mixed cryoglobulinemia [48], rheumatoid arthritis [49] or systemic lupus erythematosus [50, 51], as well as monogenic causes of immune dysregulation (e.g. *CTLA4*, *NFKB1*, and *LRBA* haploinsufficiency [39]). This B cell phenotype likely contributes to impaired humoral immunity in STAT3 GOF patients resulting in impaired Ab responses to infectious disease (hypogammaglobulinemia, recurrent common infection), as well as the accumulation of autoreactive B cell clones driving broad autoimmune pathology such as autoimmune cytopenias [39, 46]. Interestingly, increased CD21^lo^ B cells in STAT3 GOF were associated with enhanced T_FH_ cells and a skewing towards a CXCR3^+^/Th1 phenotype. A positive correlation between CD21^lo^ B cells and CXCR3^+^ T_FH_1 cells has been noted previously in CVID patients [39, 52, 53], suggestive of a mechanism underlying immune dysregulation including the production of autoAbs and immune deficiency in these patients. Future investigation could assess disease severity in the context of these biomarkers, and whether these are ameliorated following gene-targeted therapy, such as JAK inhibitors.

Our study confirmed previous reports in humans and mice of a lack of association of autoimmune pathology with diminished peripheral Tregs in STAT3 GOF Syndrome [36, 37]. Leiding *et al*., however, reported reduced CD25 expression on CD4^+^ T cells in 40% of patients [17]. Our study suggests that while the diminution in expression of CD25 on Tregs is mild (<2-fold), it is much more prominent for memory CD4^+^ T cells (3-fold), T_FH_ (7-fold). In contrast, CD25 expression is significantly enhanced as a proportion of naïve B cells (4-fold) and CD19^hi^ CD21^lo^ B cells (3-fold) in patients with STAT3 GOF Syndrome. Therefore, a dysregulation in IL-2 signaling in STAT3 GOF Syndrome could impact lymphocyte differentiation and effector function culminating in disease. Such pathogenesis may be localized within affected tissues, as has been posited in *Stat3*^GOF^ mouse models of hyper-IL-22 activity driving T_H_17-mediated skin inflammation in STAT3 GOF Syndrome [54]. Largely intact *ex vivo* cytokine secretion in peripheral CD4^+^ naïve and memory T cells from patients with STAT3 GOF Syndrome presented here may also support the notion of tissue-specific disease pathogenesis versus broad systemic autoinflammatory/autoimmune disease. However, reduced IL-9-producing CD4^+^ T cells in P1 could reveal a potential mechanism for impaired skin barrier protection and pathology that may contribute to high atopic disease burden in patients with STAT3 GOF Syndrome [55–57]. Assessment of IL-9 production and secretion in further patients would strengthen this hypothesis.

A pertinent observation in assessing lymphocyte dysregulation due to *STAT3* GOF mutations is the comparison to patients with *STAT3* LOF/DN mutations to establish the importance of STAT3 signaling in critical human immune responses. Reduced expression of the immunoregulatory molecule PD1 on T_FH_ cells in STAT3 GOF patients is a diametrically opposite observation to what is seen in CD4^+^ T cells in STAT3 LOF/DN HIES patients (i.e. enhanced PD1 expression [58]). Similarly, we previously found that *IL2RA*, encoding CD25, is a direct target of STAT3, and STAT3 LOF/DN variants impair the upregulation of CD25 expression on IL-21 stimulated naïve B cells ability of IL-21 [59]. This is consistent with aberrantly high CD25 expression on STAT3 GOF naïve B cells. These observations infer that STAT3 signaling positively and negatively regulates the expression of CD25 and PD1, respectively, on human B cells and CD4^+^ T cells. Additionally, STAT3 LOF/DN HIES patients lack memory B cells; however, the residual memory B cells have intact Ig class switching [6]. Conversely, observations from this study highlight that STAT3 GOF patients have intact proportions of CD27^+^ memory B cells, but within this memory pool, class-switch recombination to IgG and IgA isotypes is considerably abrogated. Thus, these rare patient cases provide considerable evidence that balanced STAT3 signaling is crucial for B cell differentiation to mount effective humoral immune responses in humans.

## Conclusion

Our study validated a novel *STAT3* VUS in a patient with a lengthy clinical history, using appropriate *in silico*, *in vitro,* and *ex vivo* experimental tools in line with ACMG guidelines. We performed deep immunophenotyping of five STAT3 GOF patients to investigate the cellular mechanisms of lymphocyte dysregulation responsible for disease pathogenesis. These observations serve to improve clinical management and assist validation of novel *STAT3* VUS in the future.

## Supplementary Material

uxaf005_suppl_Supplementary_Figure_S1

## Data Availability

Data included in or related to this manuscript are available on requestion to the corresponding author.
