# Supplementary material for: Functional validation of a novel STAT3 ‘variant of unknown significance’ identifies a new case of STAT3 GOF syndrome and reveals broad immune cell defects
Source: Clin Exp Immunol. 2025 Jan 21;219(1):uxaf005. doi: 10.1093/cei/uxaf005 (PMC11791529; doi:10.1093/cei/uxaf005)
Supplement: uxaf005_suppl_Supplementary_Figure_S1 [file uxaf005_suppl_Supplementary_Figure_S1.docx]

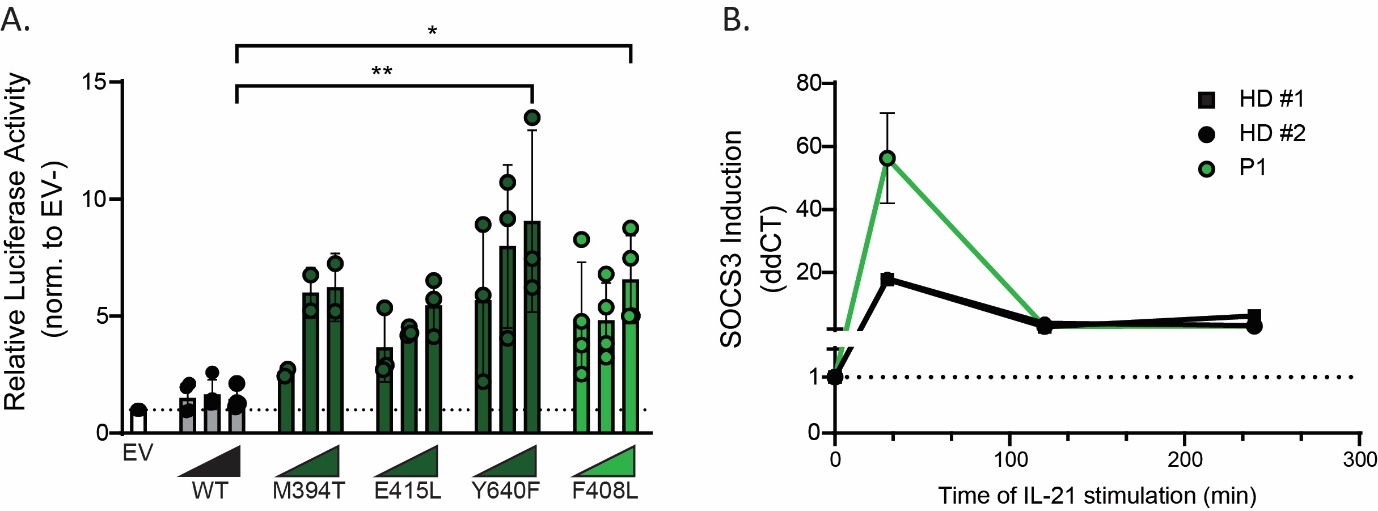


**Supplementary Figure 1:**

1. A4*^STAT3-/-^* cells were co-transfected with combinations of empty vector (EV), WT or patient variant (M394T, E415L, Y640F, F408L) *STAT3* pCMV6 vectors in addition to a firefly luciferase vector containing the M67SIE STAT3-target promoter and a constitutively expressed renilla luciferase vector. Cells were collected and lysed 24 hours later, and lysates were assessed for luciferase activity. Graph represents firefly luminescence normalized to renilla luminescence normalized to the unstimulated EV condition. Statistical significance for 25ng STAT3 dosages was assessed using a one-way ANOVA with Dunnett’s post-test (* p < 0.05, ** p < 0.01).
2. T cell blasts derived from healthy donors (HD) or P1 were rested overnight in RPMI1640 before being cultured in the absence or presence of 100ng/mL IL-21 for 30, 120 and 240 minutes. Cells were lysed, RNA extracted and transcribed to cDNA and used as template to measure *SOCS3* transcript induction using RT-qPCR. Data represents technical replicates from 1 experiment.
